# The Human Mercaptopyruvate Sulfurtransferase TUM1 Is Involved in Moco Biosynthesis, Cytosolic tRNA Thiolation and Cellular Bioenergetics in Human Embryonic Kidney Cells

**DOI:** 10.3390/biom13010144

**Published:** 2023-01-10

**Authors:** Moses Olalekan Ogunkola, Gaelle Guiraudie-Capraz, Francois Feron, Silke Leimkühler

**Affiliations:** 1 Department of Molecular Enzymology, Institute of Biochemistry and Biology, University of Potsdam, Karl-Liebknecht Strasse 24–25, Golm, 14476 Potsdam, Germany; 2 Institute of NeuroPhysiopathology (INP), CNRS, Aix Marseille University, UMR 7051, CEDEX 5, 13385 Marseille, France

**Keywords:** Moco biosynthesis, sulfite oxidase, cytosolic tRNA thiolation, 5-methoxycarbonylmethyl-2-thiouridine, H_2_S biosynthesis, cellular bioenergetics

## Abstract

Sulfur is an important element that is incorporated into many biomolecules in humans. The incorporation and transfer of sulfur into biomolecules is, however, facilitated by a series of different sulfurtransferases. Among these sulfurtransferases is the human mercaptopyruvate sulfurtransferase (MPST) also designated as tRNA thiouridine modification protein (TUM1). The role of the human TUM1 protein has been suggested in a wide range of physiological processes in the cell among which are but not limited to involvement in Molybdenum cofactor (Moco) biosynthesis, cytosolic tRNA thiolation and generation of H_2_S as signaling molecule both in mitochondria and the cytosol. Previous interaction studies showed that TUM1 interacts with the L-cysteine desulfurase NFS1 and the Molybdenum cofactor biosynthesis protein 3 (MOCS3). Here, we show the roles of TUM1 in human cells using CRISPR/Cas9 genetically modified Human Embryonic Kidney cells. Here, we show that TUM1 is involved in the sulfur transfer for Molybdenum cofactor synthesis and tRNA thiomodification by spectrophotometric measurement of the activity of sulfite oxidase and liquid chromatography quantification of the level of sulfur-modified tRNA. Further, we show that TUM1 has a role in hydrogen sulfide production and cellular bioenergetics.

## 1. Introduction

The human 3-mercaptopyruvate sulfurtransferase (MPST) (EC 2.8.1.2), also designated as TUM1 (tRNA thiouridine modification protein 1), belongs to an enzyme superfamily of proteins that contain a rhodanese-like domain (RLD) [1]. To date, the physiological function of rhodanese-like proteins is not fully understood, but rhodaneses have been linked to a wide variety of biological processes, including the detoxification of cyanide, the homeostasis of cellular sulfur in general, the participation in the degradation of L-cysteine, mitochondrial production of hydrogen sulfide (H_2_S) as signaling molecule, in addition to the biosynthesis of enzymatic cofactors, vitamins and sulfur-containing nucleic acids in tRNAs [2,3,4,5,6]. A role of the human TUM1 protein was recently suggested to be involved in the two biosynthetic pathways of tRNA thiolation of cytosolic tRNAs and Moco biosynthesis by interacting with the protein MOCS3 in humans [7]. Initially, an involvement of the yeast TUM1 protein (tRNA thiouridine modification protein) in tRNA thiomodification had been identified before [1,8]. Human TUM1 has been shown to catalyze the desulfuration of 3-mercaptopyruvate to generate an enzyme-bound hydropersulfide [9], which then transfers the persulfide’s outer sulfur atom to proteins or small molecule acceptors. MPST activity is also known to be involved in hydrogen sulfide generation, tRNA thiolation, protein urmylation and cyanide detoxification [9]. Tissue-specific changes in human MPST expression correlate with aging and the development of metabolic disease [9]. Recently, high expression of MPST has been reported in cancer tissues due to its H_2_S biosynthesis capability and a subsequent influence on cellular bioenergetics [10,11,12,13]. Deletion and overexpression experiments suggested that MPST contributes to oxidative stress resistance, mitochondrial respiratory function and the regulation of fatty acid metabolism [9].

Mammalian TUM1/MPST has been mostly studied for its physiological role in H_2_S generation in cellular sulfur metabolism [6,14,15,16]. Human MPST is mainly expressed in kidney, liver, heart and neurological cells [17]. Patients have been reported to accumulate 3-Mercaptolactate (3 ML) in the urine. 3-mercaptopyruvate is converted to 3-ML due to the deficiency of TUM1, which converts the former to pyruvate [18,19]. Patients with mercaptolactate–cysteine disulfuiduria (MCDU) have been shown to have mental retardation [19], while in some cases, mental retardation was not recorded [20]. MPST knockout mice presented an increased anxiety-like behavior [21]. In addition, investigation of MPST knockout mouse brains revealed lack of cysteine-SSH and GSSH production with 50% decreased levels of total persulfated species [22].

The interaction of TUM1 with MOCS3 suggested that TUM1 is involved in tRNA thiolation and molybdenum cofactor (Moco) biosynthesis [7]. In Moco biosynthesis, two sulfur atoms are inserted into the cyclic pyranopterin monophosphate (cPMP) backbone, forming the dithiolene group of molybdopterin (MPT), which ligates the molybdenum atom and forms the molybdenum cofactor (Moco) [23]. The conversion of cPMP to MPT is catalyzed by MPT synthase, which is composed of MOCS2A and MOCS2B [24]. For regeneration of the thiocarboxylate group at the C-terminal glycine of MOCS2A in MPT synthase [25] MOCS3, TUM1 and NFS1 are suggested to be involved [7]. Moco biosynthesis and tRNA thiolation were suggested to share the same sulfur delivery pathway composed of NFS1, TUM1 and MOCS3 [7].

In humans, there are four molybdoenzymes, namely sulfite oxidase (SO) aldehyde oxidase (AOX), xanthine oxidase (XO) and the two mitochondrial amidoxime reducing components, mARC1 and mARC2 [26]. SO catalyzes the metabolic detoxification of sulfite to sulfate within the intermembrane space of the mitochondria [27]. In mammals, SO is highly expressed in the liver, kidney and heart and has very low expression in the spleen, brain, skeletal muscle and blood [28]. Sulfite toxicity is proposed to arise from the conversion of excess sulfite to sulfite radicals in the absence and presence of oxidative stress due to deficiency in SO [29]. In humans, deficiency in sulfite oxidase deficiency, which can arise from the mutation of genes encoding for proteins responsible for Moco biosynthesis (MocoCD) [30], can lead to mental retardation, epileptic seizures, brain atrophy and (dislocated ocular lenses) [31]. Recently, it was shown that, in Moco deficient patients, highly interconnected mitochondria are present, similar to what has been shown in mouse-derived fibroblasts [32]. It has been therefore concluded that altered mitochondrial dynamics are an important contributor to the disease phenotype of sulfite oxidase deficiency in Moco-deficient patients, and it has been suggested that MoCD should be included among the mitochondrial disorders [32].

Transfer RNAs from all organisms contain modified nucleosides, which are derivatives of the four major nucleosides: adenosine (A), guanosine (G), cytosine (C) and uridine (U) [33]. tRNAs specific for lysine, glutamate and glutamine in most organisms have a 2-thiouridine derivative (xm^5^s^2^U) at the wobble uridine at position 34 [34,35,36,37]. Thiolation modifications at the wobble uridine at position 34 (U34) present in tRNA of lysine, glutamine and glutamate are suggested to be important for enhanced translation efficiency and higher stability of tRNA binding to the ribosomal A site [38]. For the thiolation and formation of the mcm^5^s^2^U in the cytosol of eukaryotes, the biosynthesis of the 5 methoxy-carbonylmethyl group of the uracil ring is required for efficient 2 thiouridine formation in the cytoplasm [8]. In humans, it was shown that the proteins MOCS3, URM1, TUM1, CTU1 and CTU2 are involved in s^2^U34 formation [39,40], while proteins of the ELP pathway synthesize the mcm5-group [41]. The ELP pathway includes the six subunits of the ELP-complex (ELP1–6) and the tRNA methyltransferase complex containing TRM9 and TRM112 [42]. The URM1 protein (ubiquitin-related modifier) was shown to have a ubiquitin-like b-grasp-fold and to contain a conserved C-terminal double glycine-motif on which a thiocarboxylate group is formed for direct sulfur-transfer to mcm^5^U34 in tRNA [25,36,43,44]. In contrast, the formation of τm^5^s^2^U34 for mitochondrial tRNALys, Gln, Glu requires different protein components compared to the ones identified in the cytosol, whereas details of the pathway are not completely resolved to date [45]. It was shown that lack of the τm^5^s^2^U modification in mitochondrial tRNALys from individuals with myoclonus epilepsy associated with ragged red fibers (MERRF) resulted in a marked defect in mitochondrial translation [46]. In this pathway, the MTU1 protein—which is a mitochondria-specific 2-thiouridylase responsible for the generation of τm^5^s^2^U in mammals—is required [47]. The sulfur is derived from mitochondrial NFS1, which is transferred via TUM1-Iso2 [7,48]. For the formation of the taurine group, the proteins GTPBP3 and MTO1 are required [47].

TUM1 catalyses the conversion of 3-mercaptopyruvate to pyruvate and a protein-bound persulfide, which is released as H_2_S [9]. Three H_2_S producing enzymes exist in humans, besides TUM1/MPST, the cystathionine-γ-lyase (CTH) and the cystathionin β-synthase (CBS). CBS and CTH are pyridoxal-phosphate (PLP)-dependent enzymes, which are differently expressed throughout tissues [49,50]. In the l-cysteine catabolism, the three enzymes are partly overlapping and complementary functions. Dysregulation of the H_2_S producing system has been linked to increased cellular dysfunction in case of diseased/stressed states [51].

We have recently shown that human interaction studies most interestingly revealed interaction of TUM1 with the only l-cysteine desulfurase of human cells NFS1, which is central for sulfur transfer in FeS biosynthesis [52], Moco biosynthesis and tRNA thiolation [53]. TUM1-Iso1 was further shown to bind MOCS3 [7], a sulfur transferase implicated in Moco biosynthesis and tRNA thiolation [39]. We hence proposed a role for human TUM1 Moco biosynthesis and tRNA thiolation. Here, we show for the first time that knockout of TUM1 in human embryonic kidney (HEK) cells impacts cytosolic tRNA thiolation as well as the Moco-dependent enzyme Sulfite oxidase (SO). Further, we show that TUM1 impacts H_2_S biosynthesis and cellular bioenergetics in HEK293T cells.

## 2. Materials and Methods

### 2.1. Materials

Sodium hydrosulfide (NaHS) obtained from (Sigma-Aldrich, Darmstadt, Hesse, Germany) was freshly dissolved in millipore water shortly before cell treatment.

### 2.2. Cultivation of Mammalian Cell Lines

HEK293T (DSMZ) cells were cultured in Dulbecco’s modified Eagle’s medium (DMEM, PAN-Biotech, Aidenbach, Bavaria, Germany) supplemented with 10% fetal bovine serum (FBS, PAN-Biotech, Aidenbach, Bavaria, Germany) and 2 mM L-glutamine (PAN-Biotech, Aidenbach, Bavaria, Germany). The cells were maintained at 37 °C and 5% CO_2_ adherently in T75 or T25 cell culture flasks (Sarstedt, Nümbrecht, North Rhine-Westaphalia, Germany) until the cells were 90% confluent. The cells were detached via trypsin/EDTA (Gibco, Life Technologies, Darmstadt, Hesse, Germany) and passaged every 3–4 days.

### 2.3. Generating TUM1 Knockout Cells with CRISPR/Cas9

The CRISPR/Cas9 method was used to generate stable TUM1 knockout cell lines. The protocol was adapted from [54]. The method is based on a complementary gRNA to target the gene of interest providing a cleavage site for the Cas9 nuclease. The cleaved DNA is repaired by error prone nonhomologous end joining (NHEJ) leading to deletions, insertions or frame-shifts preferentially resulting in loss of function mutations. gRNAs complementary to DNA near the start codon of the TUM1 gene were designed using MIT opensource (https://crispr.mit.edu/, accessed on 16 August 2017) [55]. The forward guide and the reverse guide were annealed using a standard protocol. They were constructed with *Bbs*I restriction sites to enable cloning into the pSpCas9(BB)-2A-Puro vector (https://addgene.org/crispr/, on accessed on 1 September 2017) [56]. This vector already contains the scaffolding part of the gRNA and the gene for Cas9. The resulting plasmid was transiently transfected into the HEK293T cells. Positively transfected cells were selected with puromycin. Single cells were grown into colonies and analyzed via sequencing (GATC) and immunoblotting using an α-TUM1 antibody (Abcam, Rozenburg, Amsterdam, Netherlands).

### 2.4. Immunoblotting

Whole cell lysates (50–100 μg) were separated by sodium dodecyl sulfate–polyacrylamide gel electrophoresis (SDS–PAGE) and transferred onto a polyvinylidene fluoride (PVDF) membrane (Amersham Hybond, GE Healthcare, Freiburg, Baden-Wüttemberg). Protein transfer was performed using Mini-Protean 2 Cell chambers (Bio-Rad, Kabelsketal, Saxony-Anhalt, Germany). The primary antibodies TUM1 (1:3500., Abcam, Rozenburg, Amsterdam, Netherlands), CBS (1:1000., Sigma-Aldrich, Darmstadt, Hesse, Germany), CTH(1:1000., Sigma-Aldrich, Darmstadt, Hesse, Germany), CTU1 (1:1000, Sigma-Aldrich, Darmstadt, Hesse, Germany), CTU2 (1:1000, Sigma-Aldrich, Darmstadt, Hesse, Germany), URM1 (1:500, Sigma-Aldrich, Darmstadt, Hesse, Germany), α-MOCS3 (1:4000, Abcam, Rozenburg, Amsterdam, Netherlands), α-SO (1:1000, Abcam, Rozenburg, Amsterdam, Netherlands) and α-actin (1:7500, Sigma-Aldrich, Darmstadt, Hesse, Germany) were used for protein detection together with the peroxidase-coupled secondary antibodies (α-rabbit POD, 1:10,000, Sigma-Aldrich, Darmstadt, Hesse, Germany; α-mouse POD, 1:5000, Sigma-Aldrich, Darmstadt, Hesse, Germany). The blots were developed with chemiluminescence via the Fusion SL Vilber Lourmat (peqlab, Erlangen, Bavaria, Germany) imaging system.

### 2.5. MTT Assay

MTT [3-(4,5-dimethylthiazol-2-yl)-2,5-diphenyltetrazolium bromide] is converted to insoluble purple formazan by dehydrogenases in living cells, thereby measuring the growth rate [57]. Formazan can be solubilized by isopropanol and measured spectrophotometrically. 10 × 10^3^ cells per well were seeded in a 96-well plate, and 50 μL of the MTT solution was added to each well and incubated for 3 h at 37 °C. Subsequently, 150 μL of the MTT solvent was added, and the MTT formazan was detected after 15 min at 590 nm.

### 2.6. Aconitase Activity Assay

Aconitase is an [4Fe-4S] cluster-containing TCA cycle protein that catalyzes the isomerization of citrate to isocitrate via cis-aconitate [58]. HEK293T cells were grown in T75 culture flasks until the cells were 90% confluent. The cells were harvested and lysed in nondenaturing lysis buffer [50 mM Tris-HCl and 1% NP-40 (pH 8)]. The protein concentration was determined via a Bradford assay. The aconitase activity was measured using 50 μL of cell lysate mixed with 250 μL of reaction buffer [50 mM Tris-HCl, 50 mM NaCl, 5 mM MgCl2, 0.5 mM NADP+ and 0.05 unit of isocitrate-dehydrogenase (Sigma) (pH 8)]. This was incubated for 5 min at 37 °C before the addition of 200 μL of starting buffer (50 mM Tris-HCl, 50 mM NaCl, 5 mM MgCl_2_ and 2.5 mM cis-aconitate). The reaction was followed at 340 nm by the reduction of NADPH as ICDH converted the product of aconitase. The specific activity was calculated using the extinction coefficient of NADPH (ε340 = 6220 mM^–1^) [59].

### 2.7. Sulfite Oxidase Activity Assay

The activity assay of the Moco-dependent enzyme sulfite oxidase was adapted from [60]. Cells were grown in a T75 cell culture flask until they were 90% confluent. They were harvested, and the pellet was resuspended with extraction buffer [50 mM Tris-acetate, 0.1 mM EDTA and 1% NP-40 (pH 8.5)]. The probes were vortexed and centrifuged (12,000× *g* for 15 min at 4 °C) to obtain the cell lysate. The protein concentration was determined with Bradford reagent. The enzyme activity was measured using 150 μL of cell lysate in a total reaction volume of 1 mL. The reaction buffer consisted of 800 μL of 50 mM Tris-acetate, 0.1 mM EDTA and 1% NP-40 (pH 8.5) to which 10 μL of 17 mM sodium deoxycholic acid, 5 μL of 10 μM potassium cyanide, 33 μL of cytochrome c (6 mg/mL) and 2 μL of 100 mM sodium sulfite had been added. The reduction of cytochrome c was monitored at 550 nm for 5 min. The specific activity using the extinction coefficient of cytochrome c (ε550 = 19.36 M^–1^) was calculated.

### 2.8. tRNA Extraction and Analysis

Using nucleoside separation by HPLC, it is possible to distinguish and quantify non-modified nucleosides as well as the modified nucleosides, as the mcm^5^s^2^-modification on Uridine of Lys, Gln and Glu. Nucleosides can be distinguished by the respective UV-spectra. Forty-eight hours before being harvested, the cells were seeded on three T75 cell culture flasks until the cells were 90% confluent. The cells were taken up with TriFast (Peqlab, Erlangen, Bavaria, Germany), and a 1:5 volume of chloroform was added. After centrifugation, the upper, aqueous phase was transferred into a new falcon and precipitated with 1 times the volume of isopropanol overnight at −20 °C. The samples were spun down (13,000× *g* for 1.5 h at 4 °C), and the resulting pellet was washed thrice with 70% ethanol. The pellet was dried at 37 °C for 10–15 min. The precipitated RNA was dissolved in 100 μL of 0.3 M NaOAc (pH 4.5) for 15 min at 55 °C. One hundred micrograms of total RNA per gel was separated by 10% urea–PAGE run at 200 V for 75 min. Subsequently, the gels were stained with an ethidium bromide solution, and the tRNA bands were cut out and placed in crush-n-soak buffer [50 mM sodium acetate and 150 mM sodium chloride (pH 7.0)] at 4 °C overnight to release the RNA from the gel. This was then precipitated overnight at −20 °C with a 1:1 dilution with isopropanol before being washed twice with 70% ethanol. The tRNA pellets were dried at 37 °C for 10–15 min. They were dissolved in 50 μL of 0.3 M NaOAc for approximately 15 min at 55 °C. High-performance liquid chromatography (HPLC) analysis was performed as described by [61].

### 2.9. Quantification of Moco and cPMP in HEK293T Cells

Moco and cPMP can be oxidized into their fluorescent degradation products FormA and Compound Z, respectively. These degradation products can then be eluted via QAE chromatography and quantified via HPLC [62]. Briefly, HEK293T cells were grown in T75 cell culture flasks until the cells were 90% confluent. They were harvested and resuspended in 800 μL 100 mM Tris-HCl (pH 7.2), followed by cell lysis through sonification (on 2 s, off 2 s, 20%, 45 s). The protein concentrations were determined via Bradford reagent. For both Moco and cPMP measurements, 50 μL of solution A (1063 μL of I2/KI and 100 μL of 37% HCl) was added to 400 μL of the cell lysate followed by the addition of 150 μL of the I2/KI solution. The samples were kept in the dark overnight at RT. Subsequently, 100 μL of 1% ascorbic acid was added to 400 μL of the supernatant after centrifugation. This was followed by the addition of 200 μL of 1 M Tris to change the pH to 8.3. The cPMP samples were loaded on QAE column. The FormA samples were further dephosphorylated with 30 μL of 1 M MgCl_2_ and 2 μL of fast alkaline phosphatase for 2 h. Purification of FormA and Compound Z was performed using QAE chromatography. FormA was eluted with 10 mM acetic acid from which nine fractions were collected (500 μL). The cPMP samples were eluted with 100 mM HCl collecting nine times 500 μL fractions. Thereafter, the fractions were loaded onto the HPLC system and quantified after separation on a reversed phase C18 column.

### 2.10. Measurement of Free H_2_S via Methylene Blue

Sulfides are able to convert N,N-dimethyl-*p*-phenylenediamine (DMPD) directly to methylene blue in the presence of a mild oxidizing agent (acidified ferric chloride). The methylene blue assay was employed according to [63]. Using cysteine, the reaction containing 50 mM Tris buffer pH 8.0, 2 mg cell lysate, 1 mM DTT, 10 µM PLP in a total volume of 500 µL was started with 1 mM L-cysteine and incubated at 37 °C for 1 h. For mitochondria substrate 3-mercaptopyruvate (3-MP), reaction containing CAPS buffer pH 10.5, 300 µg cell lysate, 1 mM DTT in a total volume of 500 µL was started with 1 mM 3-MP and incubated at 37 °C for 1 h. Both reactions were stopped by simultaneous addition of 50 µM DMPD and 30 mM Iron (III)-chloride. Methylene blue was formed and quantified at 670 nm against a sulfide standard curve.

### 2.11. Reactive Oxygen Species Quantification

To analyze the amount of ROS in the cells, the nonfluorescent molecule carboxy-(2′,7′-dichloro-hydrofluorescein diacetate)—which is readily converted to its highly fluorescent 2′,7′-dichlorofluorescein when the acetate groups are removed by activity of ROS—was employed and adapted from [64]. Cells were plated at 25,000 cells/well in a 96-well plate 24 h before treatment, cells were treated with (carboxy-DCFDA, Sigma-Aldrich, Darmstadt, Hesse, Germany) at a concentration of 10 μM of serum-free culture media and incubated at 37 °C for 30 min. The serum-free media containing the dye was removed and washed. Fluorescence evaluation was carried out at emission 560 nm and excitation at 488 nm.

### 2.12. Measurement of Cellular Bioenergetics

The cellular bioenergetics were measured using the seahorse extracellular flux mito stress test, which quantifies the oxygen consumption rate as described in [65]. Briefly, cells (15,000/well) were seeded on a 24-well seahorse plate in DMEM medium a night prior to analysis; the medium was replaced with seahorse medium supplemented with l-glutamine (2 mM, Gibco, Life Technologies, Darmstadt, Hesse, Germany), sodium pyruvate (1 mM, Sigma, Sigma-Aldrich, Saint Quentin-Fallavier, Lyon, France) and glucose (10 mM, Sigma, Sigma-Aldrich, Saint Quentin-Fallavier, Lyon, France). After 1 h incubation at 37 °C in CO_2_-free incubator, the oxygen consumption rate (OCR) after oligomycin (1 µM) was used to estimate the rate of ATP production. In addition, carbonyl cyanide-4-trifluoromethoxy phenylhydrazone (FCCP, 0.5 µM) was used to estimate the maximal mitochondrial respiratory capacity. The flux of electrons through complex III and I was blocked with antimycin A (0.5 µM) and rotenone (0.5 µM), respectively; any residual activity in the presence of these inhibitors was assessed as non-mitochondrial OCR. Results were normalized to the number of cells.

## 3. Results

### 3.1. Generation of HEK293T TUM1 KO Cell Lines with CRISPR/Cas9 System

**Figure 1:**
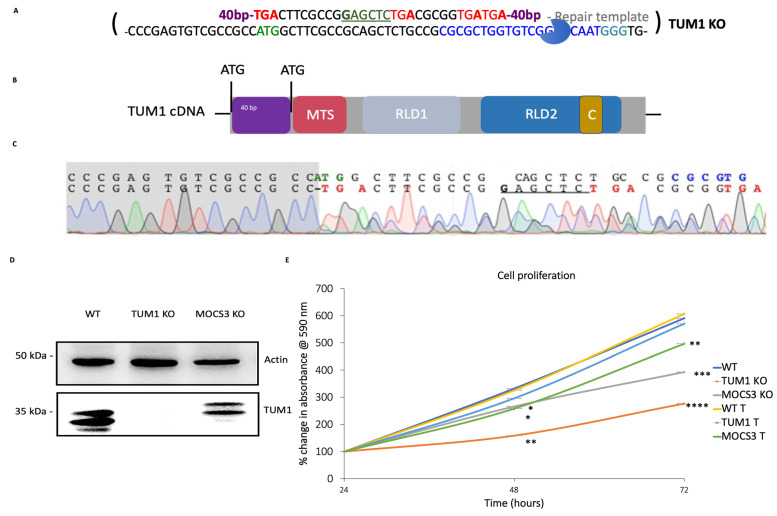
Generation of *TUM1* knockout cell lines using the CRISPR/Cas9 system. (**A**) Schematic diagram of Repair template (upper) and genomic sequence (lower) of the respective Cas9 targeting site. Homology sequences not shown (40 bp). The guide RNA (blue) with the respective PAM-site (turquoise), exon regions (yellow box), start codons (green), silent mutations (orange), restriction sites (brown), resulting in stop codons (red). (**B**) Diagrammatic representation of *TUM1* cDNA showing the ATG (start codon), MTS (mitochondria targeting sequence), RLD (rhodanese like domains) and the C terminal cysteine. (**C**) Sequence of PCR product with mutations spanning from the start codon. (**D**) Immunodetection of TUM1 in different HEK293T cell lines. The generated HEK293T CRISPR/Cas9 cell lines with knockout cells in TUM1 were analyzed for the presence of the TUM1 protein by immunodetection. (**E**) Proliferation rate with (T) and without NaHS treatment of HEK293T cell lines. MTT was added at 0.5 mg/mL to the culture media and incubated at atmosphere of 5% CO_2_ for 3 h at 37 °C. The formazan dye formed was dissolved using the permeabilization solution and measured at 570 nm. Independent samples *t*-test with SPSS was performed as indicated * *p* < 0.05, ** *p* < 0.01, *** *p* < 0.005, **** *p* < 0.001 (*n* = 3) (*n* represents number of biological replicates).

HEK293T cells were used to generate the TUM1 knockout cell line. HEK293T cells have a near triploid karyotype and contain three copies of chromosome 22 on which the TUM1 gene is localized. Co-transfection of the short guide RNA-containing Cas9-encoding plasmid with a repair-oligonucleotide that contains several stop codons at the 5′ end directs the cell repair mechanism towards homology-directed repair (HDR) and insertion of the desired genetic sequence (Figure 1A), which preferentially led to random mutations with a desired premature termination of TUM1 transcription. DNA sequencing revealed the mutations and identified several homozygous (−/−) TUM1 KO cell line (Figure 1C). Additionally, the absence of the TUM1 protein in the knockout cell line was confirmed by immunodetection, using a TUM1 specific antibodies (Figure 1D). As control, we used our MOCS3 KO cell line, reported previously [66] which shows the presence of both TUM1 isoforms, confirming that the TUM1 KO was successful and both isoforms are mutated in the produced homozygous cell line. The MTT assay was applied to analyze changes in the cell growth caused by the absence of TUM1. Here, we compared the TUM1 KO cell line to the MOCS3 KO since that cell line was shown before to have a growth defect. The results in (Figure 1E) show that the TUM1 KO strain has a higher impact on cell division in comparison to the MOCS3 KO strain. The cells were able to divide, but the growth rate was shown to be significantly reduced by 50% in the homozygous (−/−) TUM1 KO strain compared to that in wild-type cells. Treatment with exogenous H_2_S donors like NaHS in a neutral solution like water leads to about 20% H_2_S and 80% (HS-) [67]. Treatment of TUM1 KO cells with 10 μM of NaHS (as exogenous hydrogen sulfide donor) reverted the growth deficit. NaHS was applied due to previous results showing that hydrogen sulfide stimulates cellular bioenergetics at lower concentrations [68] and the known involvement of TUM1 in hydrogen sulfide production in mitochondria [69]. Conclusively, TUM1 KO cells might lower the rate of cell proliferation based on a lower hydrogen sulfide production in the mitochondria.

### 3.2. Effect of TUM1 KO on Sulfite Oxidase Activity and Moco Biosynthesis

**Figure 2:**
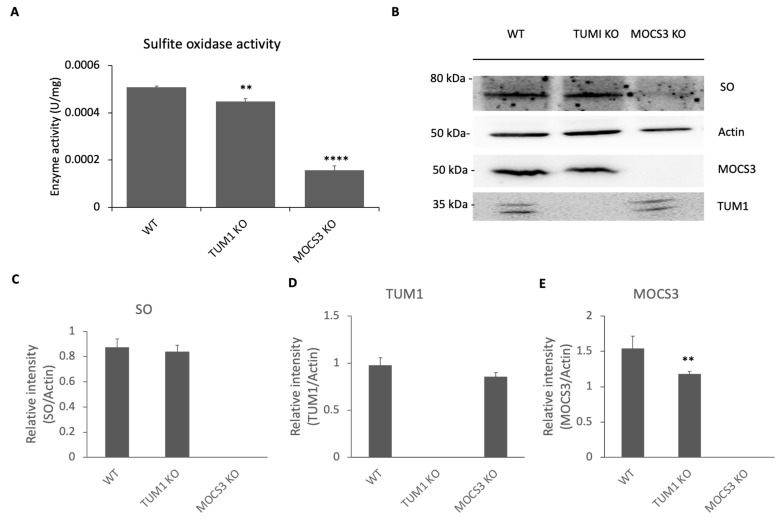
Sulfite oxidase activity in HEK293T cell lines. (**A**) Sulfite oxidase activity in wild type, *TUM1 KO*, and *MOCS3 KO*(−/−) were determined using sodium sulfite as substrate; the reaction was monitored by the reduction of cytochrome c at 550 nm for 5 min. (**B**) Immunodetection of SO, MOCS3 and TUM1 using the corresponding antibodies. 100 μg of cell lysates were loaded on a 12% SDS-PAGE and transferred to a PVDF membrane, followed by incubation with corresponding antibodies. Proteins were visualized using POD-labeled secondary antibodies. Image J intensity quantification (**C**) SO (**D**)TUM1 (**E**) MOCS3. Independent samples *t*-test with SPSS was performed as indicated ** *p* < 0.005, **** *p* < 0.001 (*n* = 3) (*n* represents number of biological replicates).

Prior studies have shown that TUM1 interacts with the l-cysteine desulfurase (NFS1) and the Molybdenum cofactor biosynthesis protein 3 (MOCS3) [7]. These two proteins are involved in two important sulfur requiring pathways; Cytosolic tRNA thiolation and Moco biosynthesis [39,53]. However, the previous studies did not investigate the role of TUM1 in these pathways in humans. To analyze a potential role of TUM1 in Moco biosynthesis, we determined the activity of the most abundant human molybdoenzyme, Sulfite oxidase, in the *TUM1 KO* cell line in comparison to the *MOCS3 KO* cell line. Again, we compared the activity to the *MOCS3 KO* cell line, which was shown before to largely reduce sulfite oxidase activity. Sulfite oxidase activity was found to be reduced to 75% of the activity of the wild type (Figure 2A) in the *TUM1 KO* cells. SO activity in *MOCS3 KO* cells a negative control was decreased to 15% of the wild type activity, in consistency with previous results [66]. Sulfite oxidase abundance was slightly reduced in *TUM1 KO* cells compared to the wild type and undetectable in *MOCS3 KO* cells (Figure 2B,C). These results confirm the past results [70] showing that SO is degraded in the absence of Moco which is evident in *MOCS3* *KO* cells and a slight reduction of SO abundance in *TUM1 KO* cells. The low amount of SO activity present in the *TUM1 KO* cells might be due to sulfur availability for MOCS3 transferred from other sulfurtransferases.

### 3.3. Effect of TUM1 KO on Moco and cPMP Levels

**Figure 3:**
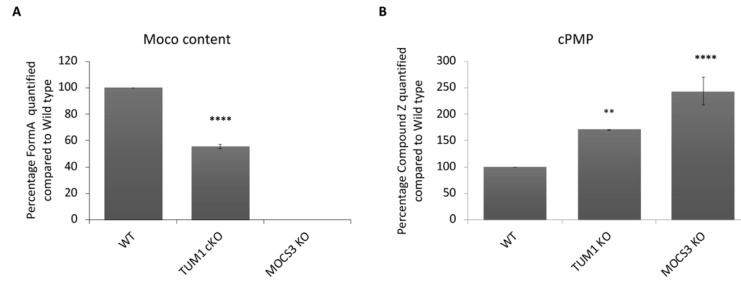
Quantification of Moco and cPMP in HEK 293T cell lines. Cells were oxidized overnight to convert Moco and cPMP to their fluorescent degradation products Form A and Compound Z, respectively. cPMP samples were separated by QAE ion-exchange chromatography and quantified by HPLC after separation on a C-18 column. 20 μL of 50% acetic acid was added to Form A samples before loading on the column. The elution of Form A and Compound Z was monitored with an Agilent 1100 series system. The fluorescence excitation at 383 nm and emission 450 nm. (**A**) Moco content after normalizing to protein concentration (**B**) cPMP content after normalizing to protein concentration. Independent samples *t*-test with SPSS was performed as indicated ** *p* < 0.005, **** *p* < 0.001 (*n* = 2).

Since sulfite oxidase activity was reduced in *TUM1 KO* cells, it was of interest to analyze the effect on Moco and cPMP production in these cells, which we compared again to the *MOCS3 KO* cell line, in which both cofactor levels were shown previously to be affected [66]. The results show that *TUM1 KO* cells had a 45% decrease in the amount of Moco compared to WT, while Moco was not detected in *MOCS3 KO* cells as reported before (Figure 3A). Additionally, cPMP accumulated 50% more in *TUM1 KO* cells compared to the WT while *MOCS3 KO* cells accumulated 120% cPMP compared to the WT (Figure 3B). These results show an effect of TUM1 for Moco production by a reduced cPMP conversion, that is the consequence of the results for the reduction of sulfite oxidase activity as shown (Figure 2). 

### 3.4. Repair of Sulfite Oxidase Activity in TUM1 KO Cells with NaHS

**Figure 4:**
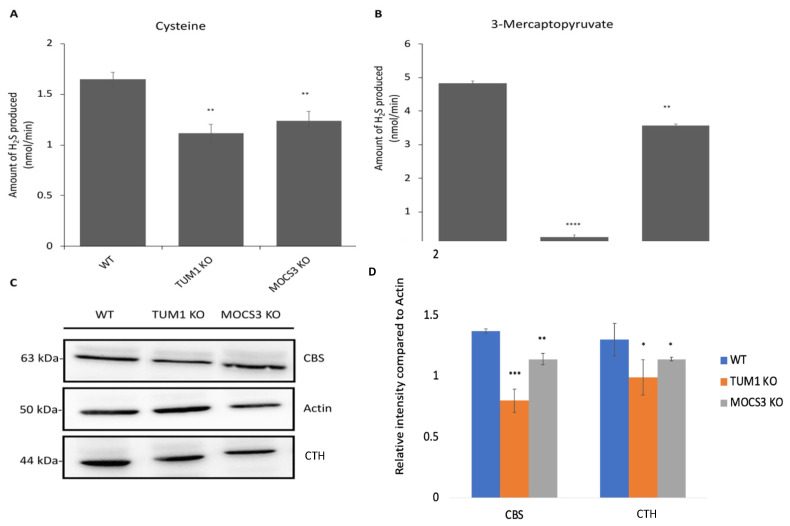
Effect of NaHS on sulfite oxidase activity in HEK293T cell lines. (**A**) Sulfite oxidase activity in WT, *TUM1 KO* and *MOCS3 KO* (−/−) were determined using sodium sulfite as substrate; the reaction was monitored by the reduction of cytochrome c at 550 nm for 5 min. (**B**) Immunodetection of SO, MOCS3 and TUM1 using respective antibodies. (**C**) Immunodetection of other H_2_S biosynthesis enzyme CBS and CTH in WT, *TUM1 KO*, and *MOCS3 KO* cells (−/−) cell lines. Proteins were visualized using POD-labeled secondary antibodies. (**D**) Image J quantification of relative intensity of blot bands of CBS and CTH compared to Actin. Independent samples *t*-test with SPSS was performed as indicated ND; no statistical difference, * *p* < 0.05, ** *p* < 0.01, *** *p* < 0.005, **** *p* < 0.001, (*n* = 3).

H_2_S has been mentioned to act via *S*-sulfhydration of target proteins. NaHS could also serve as direct sulfur donor through NaHS dissociation in neutral solution (H_2_S, HS-, S2), thereby leading to pool of sulfide [67]. The cell proliferation experiments showed that NaHS can complement the growth deficit of *TUM1 KO* cells but not of *MOCS3 KO* cells. To investigate the enzyme that is repaired by NaHS, we investigated the effect on sulfite oxidase activity. Therefore, we investigated whether the treatment of the cells with NaHS could repair the low SO activity in *TUM1* and *MOCS3 KO* cells. Here, we show that the SO activity of *TUM1 KO* cells were rescued to almost the WT level, but not the *MOCS3 KO* cells (Figure 4A). NaHS treated WT cells also showed an increase in SO activity compared to the untreated control. These data suggest that NaHS is a sulfur donor only in *TUM1 KO* cells (containing MOCS3), hence increasing the amount of Moco formed while *MOCS3 KO* cells did not respond to the NaHS treatment since MOCS3 is crucial for the sulfurtransfer reaction. One possibility is that either the transfer of sulfur from NFS1 to MOCS3 is facilitated by hydrogen sulfide by the action of TUM1. Further, it was shown previously that sulfite oxidase deficiency can be suppressed by mutation in CTH [71]. CTH was found to be present in lower amounts in TUM1 cells (Figure 4C,D); NaHS might also suppress the sulfite production from cystathionine due to the availability of hydrogen sulfide. However, this effect is not so pronounced in the *MOCS3 KO* cells since the hydrogen sulfide biosynthesis is less perturbed in *MOCS3 KO* cells compared to the *TUM1 KO* cells.

### 3.5. Effect of TUM1 KO on tRNA Thiolation

**Figure 5:**
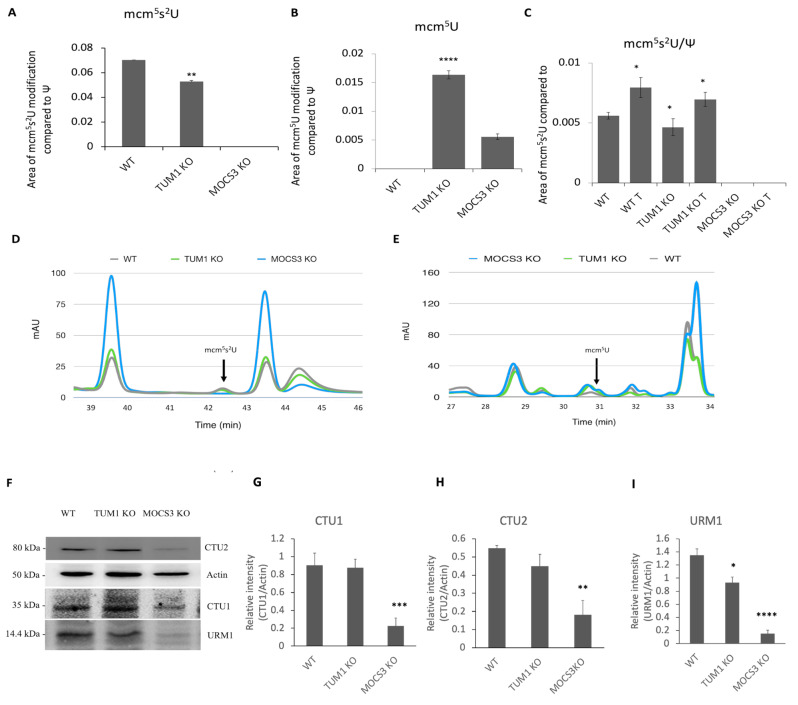
Quantification of mcm^5^s^2^U and mcm^5^U in HEK293T cell lines. Total RNAs were extracted using phenol-isopropanol precipitation. tRNAs were separated afterward from total RNA using Urea-gel. Respective tRNA were digested and corresponding nucleosides were separated and quantified by the HPLC on a C18 reversed phase column (LiCrospher 100, 5 μm particle size, 250 × 4.6 mm) (**A**) mcm^5^s^2^U (**B**) mcm^5^U (**C**) Influence of NaHS to mcm^5^s^2^U and mcm^5^U levels. T: treated with 50 µM NaHS for 16 h. (**D**) Chromatogram displaying mcm^5^s^2^U and (**E**) mcm^5^U modification at 33 and 43.4 min, respectively, of the respective cell lines using R plot (**F**) Immunoblot showing the abundance of URM1, CTU-1, CTU-2 and Actin in WT, *TUM1 KO*, and *MOCS3 KO* cells (−/−) cell lines using the respective antibodies. Proteins were visualized using POD-labeled secondary antibodies. Representative Image J intensity quantification of band intensity (**G**) CTU1 (**H**) CTU2 (**I**) URM1 Independent samples *t*-test with SPSS was performed as indicated ND; no statistical difference, * *p* < 0.05, ** *p* < 0.01, *** *p* < 0.005, **** *p* < 0.001 *(n* = 3).

TUM1 has been initially identified in a screening for mcm^5^s^2^U modified tRNA deficient mutants in yeast [8], in which TUM1 was shown to be involved, but not essential for mcm^5^s^2^U formation. Further, human TUM1 was identified to interact with NFS1 and MOCS3 in the cytosol [7], showing its involvement as a sulfur transferase for mcm^5^s^2^U formation. Therefore, we investigated the role of TUM1 on cytosolic tRNA thiolation in *TUM1 KO* cells. It is possible to distinguish and quantify non-modified nucleosides mcm^5^U from the sulfur-modified nucleosides mcm^5^s^2^U, as the mcm^5^s^2^U have a different elution time after separation on a C18 reversed phase column (LiCrospher 100, 5 μm particle size, 250 × 4.6 mm) by HPLC [61]. Quantifying the modified nucleosides, the level of mcm^5^s^2^U in *TUM1 KO* was decreased to 70% of the wild type level but undetectable in the *MOCS3 KO* cells (Figure 5A). In consistency, a higher amount of unmodified mcm^5^U was detected in *TUM1 KO* cells compared to the *MOCS3 KO* cells and below the detection limit in wild type cells (Figure 5B). This shows the role of TUM1 in sulfur transfer for cytosolic tRNA thiolation. Further, we treated the cells with NaHS to investigate the effect of extra sulfur supply on mcm^5^s^2^U formation. Here, mcm^5^s^2^U level in NaHS treated *TUM1 KO* cells were similar to mcm^5^s^2^U levels observed in control wild type cells. However, there was a similar increase in the level of mcm^5^s^2^U in the NaHS treated wild type cells (Figure 5C). This implies that H_2_S is not directly transferred to MOCS3, and the positive effect on sulfite oxidase activity is likely based on the reduced abundance of CBS and CTH (Figure 4C,D). Protein expression of CTU1, CTU2 and URM1 proteins necessary for cytosolic tRNA thiolation were also reduced in TUM1 knockout cells compared to the wild type and were largely reduced in *MOCS3 KO* cells. This suggests that the proteins might degrade in the absence of their interaction partners (Figure 5F), which is in line with previous findings where it was shown that URM1 is down-regulated under sulfur starvation conditions as other partner proteins involved in cytosolic tRNA thiolation [72]. 

### 3.6. Effect of TUM1 on H_2_S Biosynthesis

**Figure 6:**
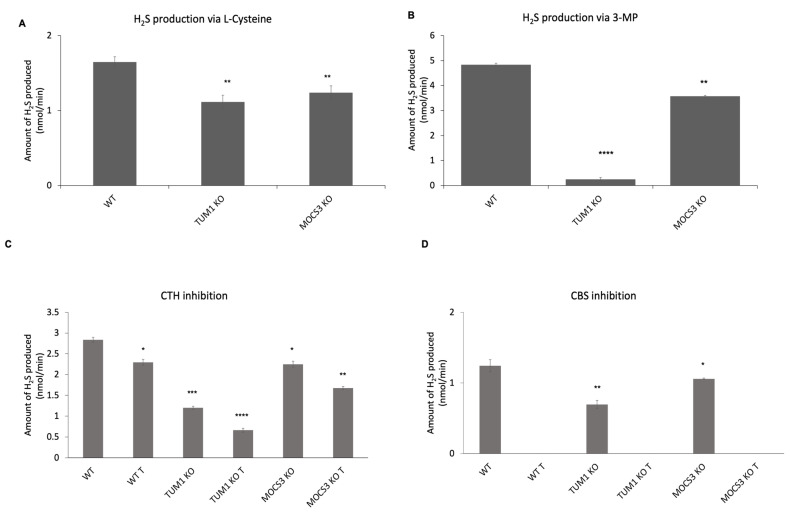
Invitro H_2_S production in HEK 293 T cell lines. Cells from WT, *TUM1 KO*, *MOCS3 KO* were lysed in NP-40 containing Tris buffer. Protein concentrations of resulting lysates were determined by the Bradford assay. H_2_S production was measured using Methylene blue assay. The activity was started by the addition of (**A**) 500 μM l-cysteine as substrate and incubated for 1 h (**B**) 1 mM 3- mercaptopyruvate as substrate and incubated for 15 min (**C**) with 2 mM propargylglycine (CTH inhibitor) treatment (**D**) with 2 mM aminooxyacetate (CBS inhibitor). Each reaction was stopped by simultaneous addition 20 mM-DMPD and 30 mM FeCl_3_ and incubated further for 20 min. The absorbance was measured at 670 nm. Independent samples *t*-test with SPSS was performed as indicated ND; no statistical difference, * *p* < 0.05, ** *p* < 0.01, *** *p* < 0.005, **** *p* < 0.001 (*n* = 3).

H_2_S is produced from sulfur-containing amino acids cysteine and homocysteine, or from 3-mercaptopyruvate, in a reaction that is catalyzed mainly by three enzymes: cystathionine beta-synthase (CBS); cystathionine gamma-lyase (CTH); and 3-mercaptopyruvate sulfurtransferase (TUM1). Of these, the first two reside in the cytosol and comprise the transsulfuration pathway. TUM1 is among the three major H_2_S producing enzymes in humans. Here, we quantified the H_2_S production in *TUM1 KO* cell lines with different substrates. When using cysteine as substrate, the total H_2_S production via all the three H_2_S producing enzymes is measured. Here, the amount of H_2_S generated in *TUM1 KO* cells was only 60% of that generated in the wild type cells. *MOCS3 KO* cells produced only 70% of H_2_S compared to the wild type (Figure 6A). When using 3-MP as substrate *TUM1 KO* cells produced only 5% of the H_2_S produced in the wild type, while the *MOCS3 KO* cells produced 75% of that of the wild type which might be consistent with the low TUM1 abundance in *MOCS3 KO* cells (Figure 6B). These results are in accordance with previous findings using different experimental approaches showing that TUM1 is the major H_2_S producing when 3-MP is available as substrate in mitochondria. Further, we validated using the methylene blue assay where we measured the activity of the H_2_S producing enzymes. To achieve this, we used the commercially available CTH and CBS inhibitors propargylglycine and aminooxyacetate (AOAA), respectively. *TUM1 KO* cells had 44% inhibition compared to 20 and 25% observed in the wild type and *MOCS3 KO* cells, respectively (Figure 6C). It was, however, impossible to measure the extent of CBS inhibition due to unselective property of the available inhibitor AOAA. This inhibitor completely inhibited the activity of all PLP dependent enzyme (Figure 6D). These results also further suggest a coping mechanism of the cell to ameliorate the effect of sulfite toxicity due to the deficiency in sulfite oxidase. CTH and CDO suppression have been found to undermine the effect of Moco deficiency in *C. elegans* [71] 

### 3.7. Effect of TUM1 on Cellular Bioenergetics

**Figure 7:**
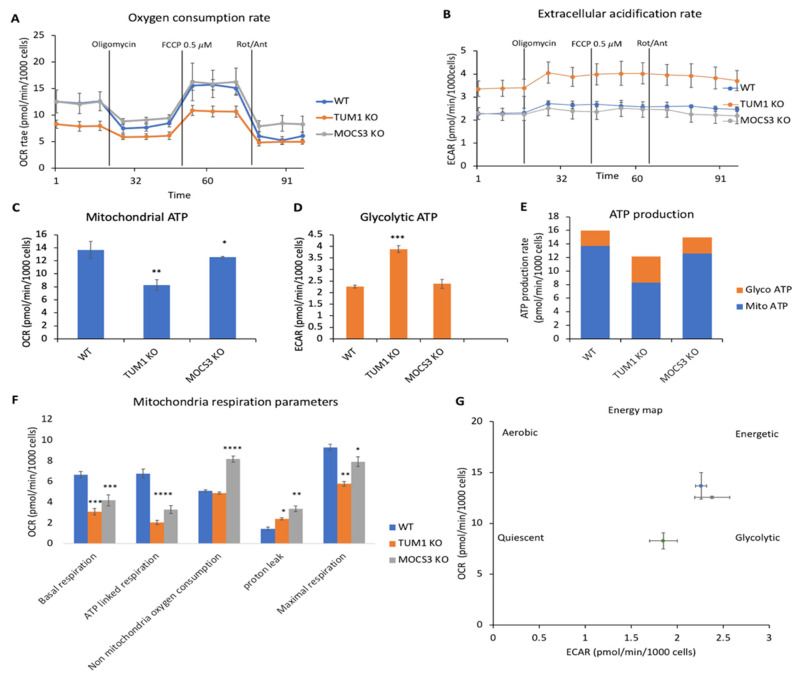
Effect of *TUM1 KO* on oxidative phosphorylation parameters in HEK 293T cells: (**A**) Oxygen consumption rate (OCR) profiles. Lines indicate the addition of specific mitochondrial stressors. Results are indicated as mean ± SD of four replicates per condition; (**B**) Representative extracellular acidification rate (ECAR). Lines indicate the addition of specific mitochondria stressors into the media. Results are indicated as mean ± SD of four replicates per condition; (**C**) analysis of mitochondrial ATP production (**D**) analysis of glycolytic ATP production (**E**) Total ATP production (**F**) analysis of oxidative phosphorylation-related bioenergetics parameters. (**G**) Energy map. All measurements were normalized to the number of cells after the seahorse measurements with Hoechst dye. Independent samples *t*-test with SPSS was performed as indicated ND; no statistical difference, * *p* < 0.05, ** *p* < 0.01, *** *p* < 0.005, **** *p* < 0.001. (*n* = 3).

H_2_S is mainly produced by cystathionine gamma-lyase (CTH), cystathionine beta-synthase (CBS) and 3-mercaptopyruvate sulfur transferase (3-MST) [73]. In contrast to the inhibitory effect of H_2_S on Complex IV, at high concentrations above 200 µM, at lower concentrations a stimulatory role of electron transport has been shown [68]. It has been suggested that H_2_S enhances the activity of FoF1-ATP (adenosine triphosphate) synthase and lactate dehydrogenase via their S-sulfhydration, thereby stimulating mitochondrial electron transport [74]. Further, H_2_S serves as an electron donor for the mitochondrial respiratory chain via sulfide quinone oxidoreductase and cytochrome c oxidase at low H_2_S levels. The latter enzyme is inhibited by high H_2_S concentrations, resulting in the reversible inhibition of electron transport and ATP production in mitochondria [75]. Mainly SQR is responsible for the oxidation of H_2_S in the mitochondria [68], from two H_2_S molecules, two disulfides (-SSH) bounds are created on the SQR, two electrons derived from two H_2_S molecules also enter the mitochondrial electron transport chain, promoting mitochondrial ATP generation [68]. Among the H_2_S producing enzymes described above, TUM1 is the major H_2_S producing enzyme in the mitochondria [73]. Therefore, we investigated the effect of TUM1 on cellular bioenergetics using the Seahorse XFe96 analyzer. Measuring the O_2_ consumption rate (OCR), an indicator of mitochondria respiration, the *TUM1 KO* cells displayed lower oxygen consumption compared to the wild-type. The *MOCS3 KO* cells also displayed a lower O_2_ consumption compared to the wild type; however, the levels were higher compared to the *TUM1 KO* cells (Figure 7A). Measuring the extracellular acidification rate (ECAR), an indicator of glycolytic respiration, *TUM1 KO* cells were higher compared to wild type, while the *MOCS3 KO* cells had similar ECAR compared to the wild type (Figure 7B). TUM1 absence led to reduced mitochondria generated ATP (Figure 7C). However, the TUM1 knockout cells produced more ATP via the glycolytic pathway(Figure 7D). Overall, ATP production was nevertheless reduced by 25% in *TUM1 KO* cells compared to wild type (Figure 7E). Data from the OCR can be used to calculate other relevant mitochondria parameters (Figure 7F). Basal respiration indicates the minimal rate of metabolism for essential maintenance function of the cell. Here, the wild type displayed higher basal respiration compared to the *MOCS3 KO* cells and *TUM1 KO* cells. However, the *MOCS3 KO* cells had higher basal respiration compared to the *TUM1 KO* cells. This suggests that the wild type cells, which proliferate much faster, require more ATP for basic metabolism and vice versa for the *TUM1 KO* cells. ATP linked respiration was higher in the wild type compared to the *TUM1 KO* cells and *MOCS3 KO* cells. However, the *MOCS3 KO* cells had more ATP linked respiration in contrast to the TUM1 knockout cells. Addition of Rotenone/Antimycin abolishes the mitochondria-linked respiration; through this, the non-mitochondria O_2_ can be measured. Here, the *MOCS3 KO* cells compared to the wild type and *TUM1 KO* cells displayed higher non-mitochondria O_2_ consumption while *TUM1 KO* cells displayed higher in comparison to the wild type. This indicates other cellular oxidative reactions like ROS, this result is in accordance with the high amount of ROS quantified in the *MOCS3 KO* cells (Figure S1). Further, there was more proton leak in the *MOCS3 KO* cells compared to the *TUM1 KO* cells and wild type, while the *TUM1 KO* cells displayed an increased proton leak in contrast to the wild type. This result implicates possible mitochondria membrane damage in the *MOCS3 KO* cells. Conclusively, the energy map shows again the dependency of *TUM1 KO* cells on the glycolytic ATP production pathway rather than the mitochondria ATP production pathway (Figure 7G). These results indicate that TUM1 impacts mitochondria bioenergetics and, consequently, ATP production, mainly in the mitochondria.

### 3.8. Effect of TUM1 KO on TCA Cycle

**Figure 8:**
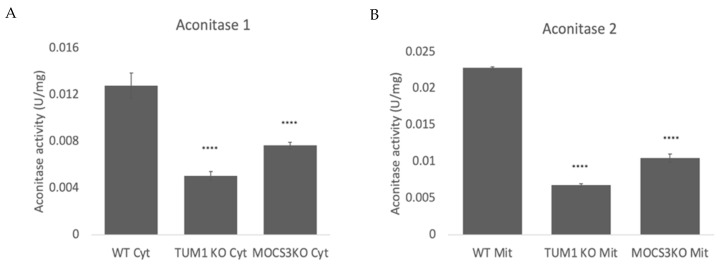
Determination of TCA cycle enzyme Aconitase in HEK293T. Cells were lysed in reaction buffer containing 0.1% NP-40. Cells were separated into cytosolic (cyt) and mitochondria (mit) fractions. The activity measurement was carried out as stated above. Enzyme activity was determined by the reduction of NAD+ at 340 nm for 3 min (**A**) Activity of Aconitase 1 (**B**) activity of Aconitase. Independent samples t-test with SPSS was performed as indicated. **** *p* < 0.001 (*n* = 3).

Aconitase is an Fe-S cluster dependent enzyme which is oxidative sensitive. There are two isoenzymes of Aconitase 1 localizing in the cytosol and Aconitase 2 localizing in the mitochondria [76]. The suppression of gene encoding for aconitase has been previously linked to reduction in cell growth and ATP [77]. Therefore, herein we investigate the effect of the two isoenzymes by measuring the aconitase activity in the cytosolic and mitochondria compartments of the cell. Measuring the cytosolic aconitase activity, there was 70% reduction aconitase activity in the *TUM1 KO* compared to the wild-type. *MOCS3 KO* also had about 50% reduction in aconitase activity compared to the wild-type (Figure 8A). In the mitochondria, the *TUM1 KO* had only 20% of aconitase activity compared to the wild-type, while the *MOCS3 KO* had only 40% of the wild-type aconitase activity (Figure 8B). These results suggest possible involvement of aconitase down regulation on the retarded growth in *TUM1* and *MOCS3 KO*.

## 4. Discussion

**Figure 9:**
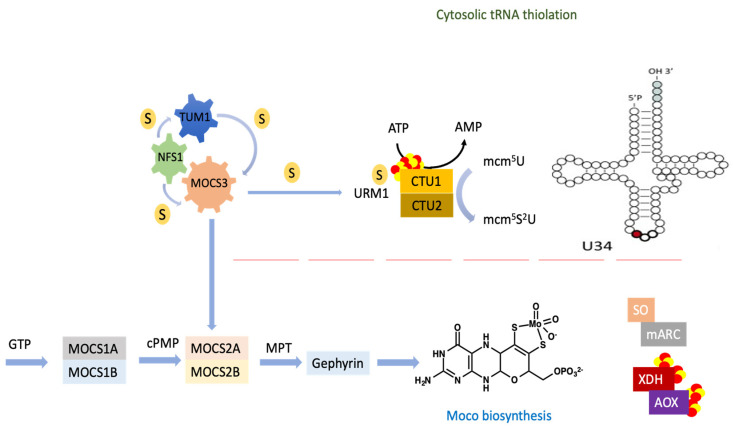
Pathway showing the transfer of sulfur from NFS1 to MOCS3 with/without the involvement of TUM1 for sulfur transfer to MOCS3. MOCS3 further activates URM1 by adenylation followed by the sulfur transfer step, which results in the formation of a thiocarboxylate group at the C-terminal Gly of URM1. URM1 subsequently interacts with the CTU1–CTU2 complex before final transfer of the sulfur to the tRNA. MOCS3 transfers two sulfur atoms to MOCS2A. MPT dithiolate is formed by incorporating two sulfur atoms from MOCS2A followed by the insertion of molybdate. This reaction is catalyzed by MPT synthase formed by the MOCS2A, MOCS2B complex.

TUM1 had been identified in yeast in a reverse genetic approach identifying genes involved in 2-thiouridine formation [8]. In that approach, five genes responsible for 2-thiouridine formation of mcm^5^s^2^U, were identified, namely NFS1, TUM1, Urm1, NCS2 and NCS6. While the other proteins were essential in that approach, TUM1 was not [8]. TUM1 contains a tandem rhodanese-like domain (RLDs). Rhodanese is a widespread and versatile sulfur-carrier enzyme catalyzing the sulfur-transfer reaction in distinct metabolic and regulatory pathway [8]. Using an in vitro sulfur transfer reaction revealed that yeast TUM1p stimulated yeast Nfs1p and accepted a persulfide sulfur atom from Nfs1p [8]. In addition, it was shown that Cys259 in RLD2 of TUM1 is responsible for efficient 2-thiouridine formation [8]. RLD1 of TUM1p, in contrast, is rather a catalytically inactive RLD, often found in various rhodanese-containing proteins because it has no conserved cysteine residue [78,79]. Those results demonstrated that NFS1 not only provides a sulfur atom to Fe/S cluster formation but also directly supplies a sulfur atom to the formation of 2-thiouridine. Yeast TUM1 therefore might act as an activator for the desulfurase of Nfs1p as well as a mediator of the persulfide from Nfs1p. Sulfur transfer for Moco biosynthesis has not been investigated in that approach or any other approach so far, based on the fact that *S. cerevisiae* is lacking Moco biosynthesis and active molybdoenzymes [80]. In this present study we report a mild effect of TUM1 on cytosolic tRNA thiolation and Moco biosynthesis leading to the proposed mechanism for sulfur transfer for the pathways (Figure 9). This similar effect was reported in cytosolic tRNA thiolation in *S.cerevisiae.* It was proposed that L-cysteine desulfurase can directly transfer the sulfur to Uba4p, the yeast homologue for MOCS3. There, TUM1p was proposed to be an activator of Nfs1p or a mediator of persulfide from the l-cysteine desulfurase. It was also shown that Uba4p could receive the persulfide directly from Nfs1p but with a reduced efficiency compared to the presence of TUM1p [8].

In general, sulfur transfer in organisms involves a network of several proteins that are conserved among organisms [81,82]. Sulfur transfer in organisms is handled by specific sulfur transferases, among which are the l-cysteine desulfurase (NFS1) rhodaneses and thiosulfate or 3-mercaptopyruvate sulfur transferases [83]. l-cysteine desulfurases generally are the initial sulfur mobilizing enzymes for many processes like FeS biosynthesis, biotin, lipoic acid biosynthesis, Moco and biogenesis of 2-thiouridines in tRNA [40]. TUM1, the 3-mercaptopyruvate sulfurtransferase in human mitochondria and the cytosol has been shown to interact with NFS1 and MOCS3 [7] proteins involved in sulfur transfer for Moco biosynthesis and cytosolic and mitochondrial tRNA thiolation [39,53]. The product of the Moco biosynthesis pathway Moco, is the active site of the four molybdoenzymes present in humans [23]. Impairment in the activity of sulfite oxidase among these enzymes has been reported to cause neurological disorders and other diseases [60]. TUM1 deficiency has been implicated to be responsible for a rare inheritable disorder known as mercaptolactate-cysteine disulfiduria (MCDU), which is associated with mental disorder [19,84] since, in *TUM1 KO* cells, sulfite oxidase activity is reduced; the mental disorder of TUM1 deficiency might be related to the symptoms of sulfite oxidase deficiency which is also characterized by neurological disorders [85]. In this report, we show that human TUM1 is involved in Moco biosynthesis and 2-thiouridine formation in addition to cellular bioenergetics through H_2_S formation. We show a decrease in the activity of sulfite oxidase activity on human embryonic kidney cell lines, in which we generated a homozygous *TUM1 KO*. The reduced sulfate oxidase activity is based on lower Moco levels in these cells, showing that TUM1 is involved in Moco biosynthesis. These results are in agreement with a previous report showing the absence of sulfite oxidase activity in *MOCS3 KO* cells [66]. It has been reported that sulfite oxidase is being degraded in the absence of Moco [70]. Hence, the reduction in sulfite oxidase activity in *TUM1 KO* cells resulted from the reduction in the amount of Moco, and reduced amounts of sulfite oxidase are likely based on the degradation of the protein in the absence of Moco. 

Recently, H_2_S has been described as acting as a sulfur donor for mitochondrial respiration [86]. H_2_S can be exogenously donated to the cells through sodium hydrosulfide (NaHS) and sodium sulfide (Na_2_S). These compounds dissociate to form HS−, and then partially binding to H^+^ to form undissociated hydrogen sulfide [87]. In previous experiments, sulfide has been shown to directly transfer sulfur in an invitro MPT biosynthesis assay [7]. In this study, we show that exogenous treatment of cells with NaHS rescues the deficit of sulfite oxidase activity in *TUM1 KO* cells. However, it has been reported that H_2_S acts by sulfurhydration of proteins in which H_2_S modulates cysteine persulfidation [88]. MPST has been shown to produce similar persulfides like cysteine persulfide (CysSSH) and GSH persulfide (GSSH) [89,90] It has also been reported that the presence of the three proteins NFS1, TUM1 and MOCS3 led to increase in Moco compared to NFS1 and MOCS3 alone [7]. Therefore, TUM1 is also involved in sulfur transfer from NFS1 to MOCS3 by enhancing the sulfur transfer to the recipient protein, a reaction that can be displaced by NaHS in *TUM1 KO* cells but not in *MOCS3 KO* cells since MOCS3 is required for Moco biosynthesis. The tRNA thiolation is also not rescued by NaHS treatment. Here, the main effect might be a higher level of oxidative stress in these cells (Figure S1) which leads to the dethiolation of tRNA (Figure S2). The dethiolated tRNA likely cannot be repaired by NAHS. Further, CTH level in *TUM1 KO* cells and *MOCS3 KO* cells were found to be reduced. This suggests a coping mechanism by the cell to reduce the toxic effect of sulfite due to Moco deficiency. CTH is known to catalyze the formation of cysteine from Cysthathionine and also the forward production of hydrogen sulfide from cysteine [91]. Increased production in H_2_S could also add up to the sulfite pool in the cell. In close cooperation with SQR, glutathione persulfide (GSSH) is generated since it is the preferred physiological co-substrate [92]. Then PDO converts GSSH + oxygen into sulfite + GSH. Sulfite is then converted (together with another GSSH molecule) into thiosulfate + GSH by rhodanese [92].

Cytosolic tRNA thiolation is an important pathway in humans that ensures proper and efficient translation [93]. Perturbation of this process has been shown to cause susceptibility to oxidative stress conditions and could also lead to misreading and misfolding of proteins [8]. Formation of mcm^5^s^2^U is dependent on the URM1 pathway, also involving MOCS3 and CTU1 and CTU2. Our results show that human TUM1 impacts cytosolic tRNA cmcm^5^s^2^U thiolation. The yeast homolog TUM1p has been described to participate in cytosolic tRNA thiolation for the mcm^5^s^2^U modification of the wobble uridine at position 34 (U34) in lysine, glutamine and glutamate [8]. In yeast, it has been reported that l-cysteine desulfurase relays the sulfur to TUM1 or directly transfers it to the Uba4 for the biogenesis of 2-thiouridine, which makes TUM1 important, but not essential for efficient transfer of sulfur [8]. In accordance to the data from yeast, human TUM1 is required but not essential for the efficient transfer of sulfur for the formation of cytosolic mcm^5^s^2^U modification, and the levels in the *TUM1 KO* are only reduced to 60% of wild type levels. We further show that CTU1, CTU2 and URM1 protein levels are reduced *TUM1 KO* cells, a reduction that is also observed in *MOCS3 KO* cells. This correlates with previous data on URM1, in which down regulation of URM1 under sulfur starvation conditions was reported in yeast [72]. We further observed an increase in the amount of mcm^5^s^2^U after exogenous supplementation of cells with NaHS, thereby repairing the loss of TUM1 function in *TUM1 KO* cell (Figure 3).

In this present research, we show retarded growth in *TUM1* and *MOCS3 KO* and subsequently demonstrated that the retarded growth rate could be due to a reduced level of H_2_S production compared to the wild-type or the reduced activity of the mitochondria aconitase. The TCA cycle is essential for the production of energy in form of ATP for maintaining high energy demanding physiological functions like cell growth [94]. Recently, it has been reported that the suppression of TCA cycle enzymes using genetic silencing siRNA showed that aconitase 2 affected the growth of CHO cells [77]. The other TCA cycle enzymes did not have significant effect on the growth rate and viability of the CHO cells. A reduction in aconitase 1 and 2 was observed in *TUM1 KO* cells as well as retarded cell growth. However, complementation of *TUM1 KO* cells with exogenous NaHS complemented for the retarded growth rate. The retarded growth can therefore be ascribed to the cellular bioenergetics influencing the aconitase activity.

Cysteine is the major H_2_S biosynthesis substrate in humans using transsulfuration pathways [95]. H_2_S is long viewed as toxic gas, and environmental hazard is emerging as a biological mediator with remarkable physiological and pathophysiological relevance [96]. TUM1 was described to be involved in H_2_S production and signaling in the mitochondria [97]. Recently, H_2_S has been described to act as a sulfur donor for mitochondrial respiration [86]. H_2_S is also produced in the cytosol by cystathionine β-synthase (CBS) and cystathionine γ-lyase (CTH) [97]. In addition, CTH and CBS are mainly located in the cytosol but translocate into mitochondria under oxidative conditions [98]. TUM1 has been previously characterized to have two distinct isoforms TUM1 Iso1 which localizes only in the cytosol and TUM1 Iso2 localizing both in the cytosol and mitochondria [7]. In the mitochondria, 3-mercaptopyruvate sulfurtransferase (3-MPST) produces H_2_S from 3MP (3-mercaptopyruvate), which is generated by CAT (cysteine aminotransferase) from l-cysteine and α-ketoglutarate [2,17]. We demonstrated that TUM1 has significant involvement in H_2_S biosynthesis in both cytosol and mitochondria, following the reduction of free H_2_S biosynthesis using cysteine and 3-MP as substrate. H_2_S produced in the vicinity of the mitochondria, in cooperation with the sulfide-oxidizing unit (SOU), stimulates and balances mitochondrial electron transport [68]. The SOU is constituted of mitochondrial membrane-bound sulfide quinone reductase (SQR) and two other enzymes the sulfur dioxygenase (ETHE1, also called dioxygenase ethylmalonic encephalopathy) and the thiosulfate sulfurtransferase (TST, also known as one isoenzyme of the rhodanese), ensuring the final oxidation of the two disulfides consuming molecular oxygen and water [68]. SQR is responsible for the oxidation of H_2_S in the mitochondria [68], from two H_2_S molecules, two disulfides (-SSH) bounds are created on the SQR, two electrons derived from two H_2_S molecules enter the mitochondrial electron transport chain, promoting mitochondrial ATP generation. Although higher concentrations of H_2_S can also inhibit Complex IV, thereby inhibiting mitochondrial potential [68]. However, in ∆MST mouse models, the activity of the complex IV was found to be similar to the WT [99]. It has been reported that the silencing of 3-MST in liver cell cultures led to reduced bioenergetics and concomitant stimulation by 3-MP, at low concentrations [96]. In ∆MST mouse, the expression of CTH was found to be reduced compared to the wild type mouse. Here, we also reported similar reduction in the abundance of CTH in the *TUM1 KO* cells [99]. The effect of the other two H_2_S producing enzymes CBS and CTH could not be annulled since H_2_S can diffuse into the mitochondria [100]. However, studies reported that CTH did not affect the cellular bioenergetics in smooth muscle cells under normal conditions [98]. It was also reported that CTH deficiency promotes ETC in blood cells [101]. Mice deficient in CTH were also shown to have decreased mitochondria biogenesis. These alternating outcomes suggest that the effect of H_2_S enzymes may defer between different tissues and complexity. We reported significant reduction in the total ATP production in the *TUM1 KO* cells and were more dependent on the glycolytic ATP production pathway rather than the mitochondria ATP production pathway. High proton leak was observed in the *TUM1* and *MOCS3 KO* cells, causing damage to the mitochondria membrane. Sulfite has been reported to alter the mitochondria in molybdenum cofactor deficiency [32], following the sulfite dependent increase in ROS with concordant decrease in ATP [102]. Non mitochondria O_2_ consumption is caused by ROS production and other oxidative reactions [103]. We observed that non-mitochondria O_2_ consumption is increased in both *MOCS3* and *TUM1 KO* and an increase in ROS level (Figure S1). Furthermore, inhibition of glutamate dehydrogenase was linked to ATP production via sulfite accumulation [102]. In accordance to this, a decreased rate of glutamate oxidation indicating low level of glutamate dehydrogenase was observed in *TUM1 KO* although not so pronounced in the *MOCS3 KO* (Figure S3). Therefore, the lower ATP level in the *TUM1 KO* compared to the *MOCS3 KO* could be a combined alteration in H_2_S biosynthesis and glutamate dehydrogenase inhibition (Figure S3). This could also be explained by the low expression of CTH and CBS in the *TUM1 KO* cells but not less pronounced in the *MOCS3 KO*. In *C. elegans*, suppression of CTH and CDO has been shown to alleviate the effect of Moco deficiency on growth in the cell. CTH and CDO through cysteine produces sulfite, thereby inhibiting growth rate [71]. Therefore, TUM1 is not only important for Moco biosynthesis and 2-thiouridine formation but also for cellular respiration and ATP production. The fact that we see an impairment of cellular bioenergetics, ATP production in mitochondria in addition to the reduced sulfite oxidase activity in *TUM1* and *MOCS3 KO* cells, fits well into what has been observed in fibroblasts from sulfite oxidase KO patients, where an impaired ATP production and an SO_3_^2−^ induced mitochondrial fragmentation has been observed [32]. Therefore, our results fit well into what has been observed before in cells with higher SO_3_^2−^ levels based on a reduced sulfite oxidase activity where decreased ATP levels, impaired cellular respiration, inhibition of glutamate dehydrogenase and malate dehydrogenase were reported [102].

## Data Availability

Not applicable.

## Supplementary Materials

The following supporting information can be downloaded at: https://www.mdpi.com/article/10.3390/biom13010144/s1, Figure S1: Quantification of reactive oxygen species in HEK293T; Figure S2: Effect of oxidative stress on tRNA thiolation. Figure S3: Effect of oxidative stress on tRNA thiolation.
