# Supplementary material for: The Human Mercaptopyruvate Sulfurtransferase TUM1 Is Involved in Moco Biosynthesis, Cytosolic tRNA Thiolation and Cellular Bioenergetics in Human Embryonic Kidney Cells
Source: Biomolecules. 2023 Jan 10;13(1):144. doi: 10.3390/biom13010144 (PMC9856076; doi:10.3390/biom13010144)
Supplement: Supplementary file 1 [file biomolecules-13-00144-s001.zip › biomolecules-2094583-supplementary.pdf]

biomolecules

Supplementary data

Results

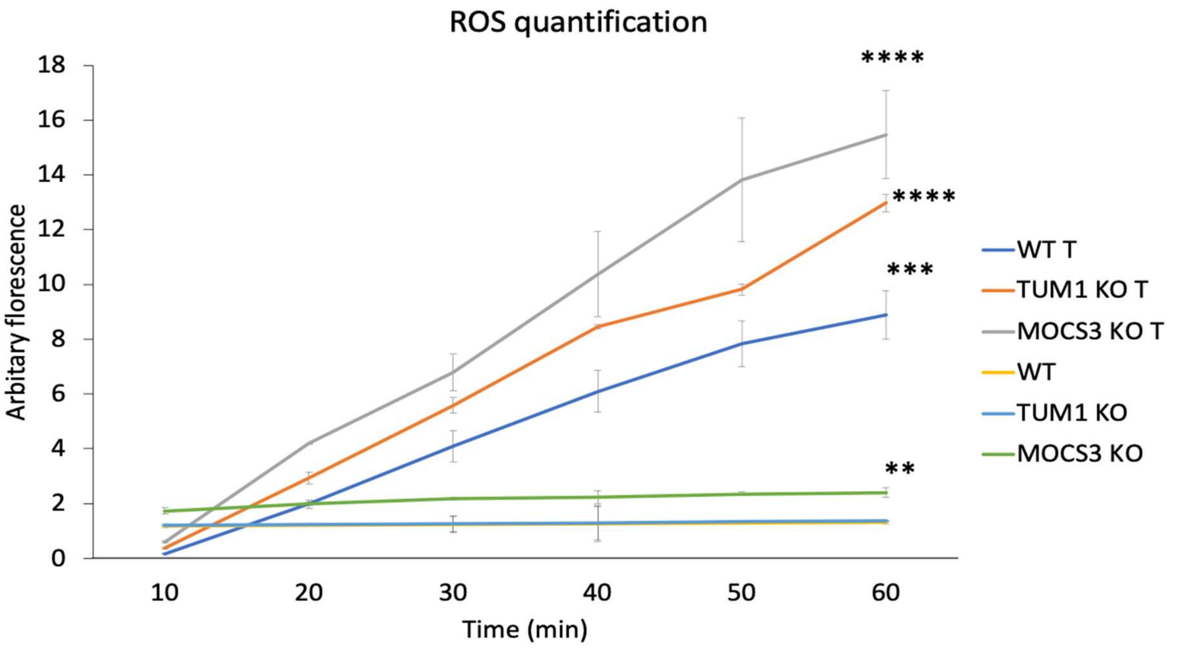

Figure S1. Quantification of reactive oxygen species in HEK293T. After 1 h 50 µM H_2O_2 treatment ROS generated in WT and *TUM1* KO were quantified using the carboxy-DCFHDA. Mean change in fluorescence was plotted for WT, *TUM1* KO and *MOCS3* KO. T represents cell lines treated with 50 µM H_2O_2. ($n = 4$; *n* represents number of biological replicates). ($n = 4$). Independent samples t-test with SPSS was performed as indicated ND; no statistical difference, *; $p < 0.05$, **; $p < 0.01$, ***; $p < 0.005$; ****; $p < 0.001$.

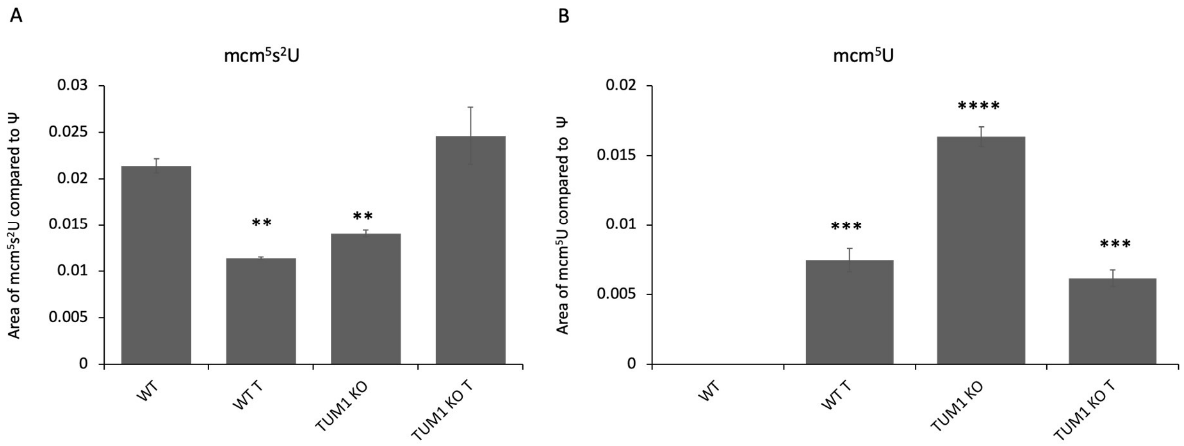

Figure S2. Effect of oxidative stress on tRNA thiolation. Cells were treated with 50 µM H_2O_2 and total RNAs were extracted using phenol-isopropanol precipitation. tRNAs were separated afterward from total RNA using Urea gel. T represents cell lines treated with 50 µM H_2O_2. Respective tRNA were digested and corresponding nucleosides were analyzed on the HPLC for (**A**) mcm^5s^2U (**B**) mcm^5U ($n = 3$). Independent samples t-test with SPSS was performed as indicated ND; no statistical difference, *; $p < 0.05$, **; $p < 0.01$, ***; $p < 0.005$, ****; $p < 0.001$.

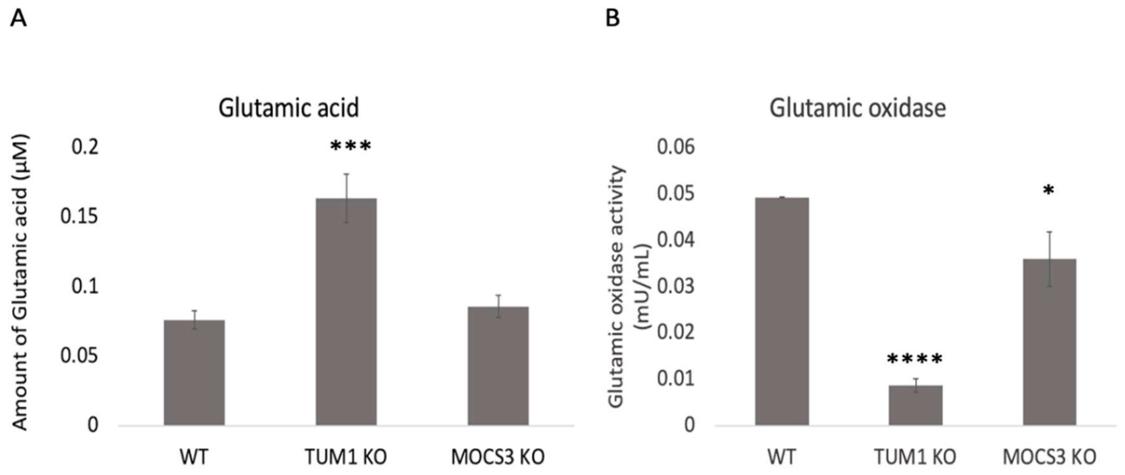

Figure S3. Quantification of glutamic acid and glutamic oxidase activity in HEK 293T. Briefly 60 µg of cell lysate was diluted in reaction buffer and mixed with 1:1 Amplex red reagent. The excitation and emission was measured for 1 hour at 571 and 585 nm respectively (**A**) amount of glutamate (**B**) glutamic oxidase activity. ($n = 3$). Independent samples t-test with SPSS was performed as indicated ND; no statistical difference, *; $p < 0.05$, **; $p < 0.01$, ***; $p < 0.005$, ****; $p < 0.001$.
